# Cardiovascular Imaging for the Early Detection of Cardiotoxicity from Emerging Cancer Therapies: Mechanistic Insights Across the Pediatric and Adult Spectrum

**DOI:** 10.3390/jpm16060322

**Published:** 2026-06-15

**Authors:** Camilla Calvieri, Isabella Leo, Jessica Ielapi, Giulia Guglielmi, Gian Luca Ragazzoni, Vincenzo D’Ambrosio, Leonie Luedke, Sara Moscatelli

**Affiliations:** 1 Haematology Unit, Department of Precision and Translational Medicine, University of Rome “La Sapienza”, Via Benevento 6, 00161 Rome, Italy; 2 Department of Experimental and Clinical Medicine, “Magna Grecia” University, 88100 Catanzaro, Italyvincenzo.dambrosio@studenti.unicz.it (V.D.); 3 Cardiovascular Research Center, Magna Graecia University, 88100 Catanzaro, Italy; 4 Pediatric and Adult Congenital Heart Centre, IRCCS-Policlinico San Donato, 20097 Milan, Italy; 5 Sports Cardiology and Rehab Unit, Department of Medical Biotechnologies, University of Siena, Viale Mario Bracci, 16, 53100 Siena, Italy; 6 Centre for Inherited Cardiovascular Diseases, Great Ormond Street Hospital, London WC1N 3BH, UK; 7 Centre for Paediatric Inherited & Rare Cardiovascular Disease, Institute of Cardiovascular Science, London WC1N 1DZ, UK

**Keywords:** cardio-oncology, cardiotoxicity, cancer therapy-related cardiac dysfunction, echocardiography, cardiac magnetic resonance, pediatric oncology, radiotherapy, targeted therapies, artificial intelligence

## Abstract

Cancer therapies have significantly improved survival but are frequently limited by cancer therapy-related cardiovascular toxicity (CTR-CVT). Cardiovascular imaging plays a central role in baseline risk stratification, surveillance during therapy and long-term follow-up. Transthoracic echocardiography (TTE) remains the first-line imaging modality; however, conventional parameters such as left ventricular ejection fraction (LVEF) often fail to detect early myocardial injury. Myocardial deformation imaging, particularly global longitudinal strain (GLS), has emerged as a sensitive marker of subclinical dysfunction across multiple cardiotoxic phenotypes. Cardiac magnetic resonance (CMR) further enhances diagnostic accuracy through tissue characterization techniques, enabling the detection of myocardial edema, inflammation, and fibrosis before overt functional decline. Different anticancer therapies induce distinct pathophysiological mechanisms of injury, each associated with characteristic imaging patterns. Emerging imaging biomarkers and multimodality approaches may improve early detection, the spatial characterization of myocardial injury and individualized surveillance strategies. Pediatric patients represent a uniquely vulnerable population due to their myocardial immaturity, altered pharmacokinetics and prolonged post-treatment life expectancy, resulting in a higher cumulative lifetime cardiovascular risk. In conclusion, a mechanism-based multimodality imaging approach integrating echocardiography, CMR and emerging data-driven technologies is essential to optimize early detection, risk stratification and long-term cardiovascular outcomes in both adult and pediatric cardio-oncology populations.

## 1. Introduction

### 1.1. The State of the Art of Cardio-Oncology

Antineoplastic agents have significantly improved the life expectancy of many patients affected by cancer. However, it has been noticed that many of these drugs can lead to cardiovascular toxicity (CTR-CVT) consistent with a broad spectrum of cardiovascular complications that includes five major categories: cardiac dysfunction (cardiomyopathy and heart failure (HF)), myo-pericarditis, vascular toxicity, hypertension, and arrhythmias with or without QTc prolongation [1,2,3,4].

Traditionally, these adverse events have been associated with anthracyclines, tyrosine kinase inhibitors (TKIs), anti-human epidermal growth factor receptor 2 (HER2) agents and inhibitors of the vascular endothelial growth factor (VEGF); however, newer therapeutic classes, such as immune checkpoint inhibitors (ICIs), have been shown not to be without these risks either.

Indeed, CTR-CVT represents one of the most serious adverse effects of anticancer therapy, leading to increased morbidity and mortality, making cardiac surveillance as essential as monitoring for cancer recurrence [5]. Cardiac injury can appear early (during therapy), late (within the first year after treatment), or very late (beyond one year post-therapy) [6].

Therefore, early risk identification, structured surveillance, and informed therapeutic decisions are essential to balance oncologic efficacy with cardiovascular safety.

The 2022 European Society of Cardiology (ESC) cardio-oncology guideline identifies baseline cardiovascular risk stratification as the critical first step in preventing CTR-CVT and recommends the use of the Heart Failure Association–International Cardio-Oncology Society (HFA-ICOS) risk assessment tool [7]. Based on these parameters, patients are stratified into low-, moderate-, high-, or very-high-risk groups, according to the estimated risk of CTR-CVT [8].

Although some real-world studies support this method’s utility in predicting moderate to severe or symptomatic cancer therapy-related cardiac dysfunction (CTRCD), it may underestimate risk when mild or subclinical forms of cardiotoxicity are present [9,10,11]. Nevertheless, this integrated approach is a good base for tailoring surveillance and guiding the early implementation of cardioprotective therapies, such as ACE inhibitors (ACEis), angiotensin receptor blockers (ARBs), β-blockers, angiotensin receptor–neprilysin inhibitors (ARNIs), and SGLT2 inhibitors [2,12,13].

### 1.2. Why Is Imaging Paramount in Detecting Subtle Changes?

Cardiovascular imaging is pivotal for risk assessment, surveillance, and evaluation of new cardiac symptoms [8,14]. Transthoracic echocardiography (TTE) remains the first-line modality [14], with the integration of left ventricular ejection fraction (LVEF) and global longitudinal strain (GLS) enabling the classification of CTRCD into three categories [7]. However, LVEF alone is limited by its dependence on loading conditions and its delayed reduction, typically occurring 3–12 months after treatment, when myocardial damage may already be advanced. For this reason, GLS should be systematically incorporated at baseline and during follow-up to detect early subclinical dysfunction [9,15,16,17,18]; in addition, atrial strain is emerging as a complementary parameter that may further improve the early detection of CTRCD [19,20,21]. Cardiac magnetic resonance (CMR) is a key complementary modality and should be used when TTE is inconclusive or when specific conditions such as myocarditis, coronary artery disease, Takotsubo syndrome, pericardial disease or valvular abnormalities are suspected. Notably, tissue abnormalities may be identified before changes in ventricular volumes or LVEF become evident [20,21,22,23].

## 2. Mechanisms of Cancer Therapy-Related Cardiotoxicity and Imaging Correlates

The comprehension of the mechanisms underlying cardiotoxic effects is essential to enhance early detection and timely intervention. Despite significant progress, elucidating the pathways underlying chemotherapy-related cardiotoxicity remains challenging, as cardiac injury results from multiple interacting mechanisms and may emerge years after treatment completion [24,25,26]. Traditionally, chemotherapeutic agents have been categorized as type I or type II, reflecting irreversible or reversible damage, respectively [27]. However, this dichotomous classification does not adequately capture the cardiotoxic profiles of many antineoplastic agents, which often exhibit mixed or atypical patterns of myocardial damage. Recent paradigms emphasize that pre-existing systemic pathologies, such as chronic inflammation, act as a primary catalyst that worsens drug-induced cardiac stress in CTRCD [28]. Systemic chronic inflammation modulates crucial signaling cascades, releasing elevated levels of pro-inflammatory cytokines such as interleukin-6 (IL-6), tumor necrosis factor-alpha (TNF-alpha), and interleukin-1beta (IL-1beta), which crosstalk with metabolic and electrophysiological pathways within cardiomyocytes, driving pathological changes and precipitating downstream functional decline and heart failure development [28].

### 2.1. Anthracyclines

**Figure 1:**
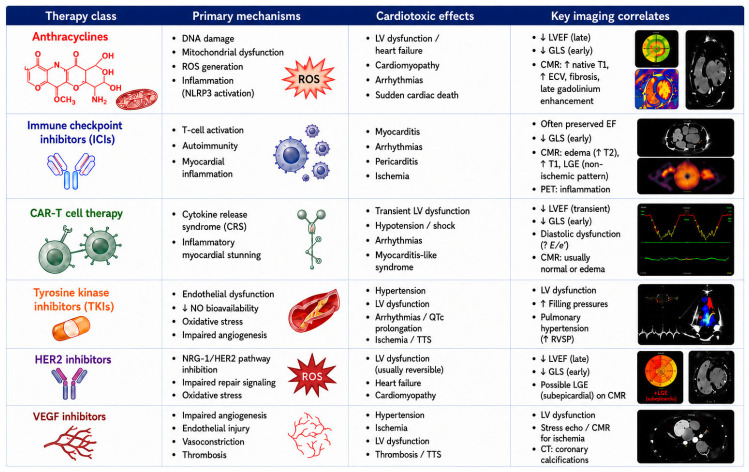
Mechanisms of cancer therapy-related cardiovascular toxicity (CTR-CVT) and corresponding multimodality imaging findings. Different anticancer therapies induce distinct patterns of myocardial injury, including oxidative stress (ROS), mitochondrial dysfunction, immune-mediated inflammation, endothelial injury, and fibrosis. These mechanisms are associated with characteristic imaging abnormalities detectable through echocardiography, cardiac magnetic resonance (CMR), and nuclear imaging techniques. Global longitudinal strain (GLS) reduction may identify early subclinical dysfunction, while CMR tissue characterization techniques, including native T1/T2 mapping, extracellular volume (ECV), and late gadolinium enhancement (LGE), provide assessment of myocardial edema, inflammation, and fibrosis.

Anthracyclines, particularly doxorubicin, remain a cornerstone of anticancer therapy but are associated with well-established dose-dependent cardiotoxicity. From a pathophysiological perspective, anthracycline-induced cardiac injury is primarily mediated by DNA damage and mitochondrial dysfunction, leading to excessive reactive oxygen species (ROS) production, impaired ATP synthesis, and the activation of apoptotic pathways [29,30,31,32,33] (Table 1, Figure 1). The activation of inflammatory cascades, including the NLRP3 inflammasome, further amplifies myocardial injury, promoting cardiomyocyte loss, microvascular damage, and progressive adverse remodeling [33,34,35]. Furthermore, the mechanistic target of rapamycin (mTOR) signaling network serves as a critical upstream hub in doxorubicin (DOX)-induced myocardial injury. Systematic in vivo evidence underscores that DOX administration fundamentally disrupts cardiac homeostatic signaling, predominantly through the suppression of the survival-promoting PI3K/Akt/mTOR pathway, thereby depriving cardiomyocytes of vital growth signals and accelerating apoptotic signaling cascades [36].

Clinically, this translates into a spectrum of cardiotoxic effects ranging from asymptomatic LV dysfunction to overt heart failure, dilated cardiomyopathy, arrhythmias, and, in severe cases, sudden cardiac death [37,38,39] (Figure 1). Unlike other forms of cancer therapy-related cardiotoxicity, anthracycline-induced damage is typically progressive and often irreversible, particularly at higher cumulative doses.

In this context, cardiac imaging plays a pivotal role in both early detection and longitudinal monitoring. While LVEF remains the standard, pooled data confirm its insensitivity to early myocardial insult, as it often remains within normal limits (>50–55%) for up to 6 months post-chemotherapy [40]. Conversely, speckle tracking echocardiography-derived GLS demonstrates robust diagnostic accuracy (AUC = 0.818), identifying functional reduction as early as 1 to 3 months after treatment initiation [40] (Figure 1). In a prospective study of 114 HER2-negative breast cancer patients treated with anthracycline-based chemotherapy, it was shown that, despite a preserved LVEF, up to 38% of patients exhibited a ≥10% reduction in GLS, and 47% had a reduced absolute GLS to <16% [41] at 12 months. Notably, a baseline GLS ≥ 20.5% had a sensitivity and specificity of 79% and 87% of maintaining a normal GLS at follow-up, even in the presence of a significant relative decline [41]. Beyond mechanical deformation, a large meta-analysis indicated that anthracycline exposure is associated with an immediate, significant elevation in native myocardial T1 relaxation times compared to both baseline and healthy cohorts [42]. Several studies confirmed that anthracycline therapy significantly increases myocardial extracellular volume (ECV) in cancer survivors with preserved LVEF. Specifically, significant post-treatment ECV elevations have been reported (29.5 ± 4.5% vs. 27.4 ± 2.3%, *p* = 0.006) [43] (Table 1, Figure 1). Prospective CMR feature-tracking studies reveal that significant impairment in RV GLS and biatrial reservoir strain occurs more frequently than conventionally defined left ventricular cardiotoxicity [44]. Taken together, these findings support a paradigm shift toward integrating both speckle tracking GLS and CMR parametric mapping into routine cardio-oncology surveillance to exploit the early “window of vulnerability” and guide timely cardioprotective interventions in anthracycline-treated patients.

### 2.2. Immune Checkpoint Inhibitors

ICIs have drastically changed the treatment of some malignant tumors by blocking key immune checkpoints such as cytotoxic T-lymphocyte antigen 4 (CTLA-4) and anti-PD/PD-L1 (programmed death receptor and receptor ligand-1) [45,46]. This blockage prompts the immune activation of T-cells against cancer but can, in turn, cause immune-related adverse events in off-target organs [47] (Table 1, Figure 1). The therapeutic blockade of co-inhibitory pathways (e.g., PD-1/PD-L1, CTLA-4) triggers a metabolic conflict in the cardiovascular microenvironment. While activated T-cells shift to glycolysis to drive proliferation and cytokine release, neighboring cardiomyocytes experience impaired fatty acid beta-oxidation. This metabolic crosstalk initiates a damaging intracellular cascade via the AMPK/mTOR axis, resulting in mitochondrial dysfunction, ROS accumulation, and NLRP3 inflammasome activation, which ultimately leads to cardiomyocyte pyroptosis and myocarditis [47]. From a pathophysiological perspective, ICI-related cardiotoxicity is primarily driven by immune-mediated myocardial inflammation resulting from the loss of immune tolerance and T-cell cross-reactivity, leading to lymphocytic infiltration, myocardial edema, and cardiomyocyte injury [48,49,50,51].

A recent study found an overall prevalence of 4.2% of cardiac ICI-related adverse events, which is almost doubled when dual ICI therapies are compared to monotherapy (5.8 vs. 3.1%, respectively) [50]. Myocarditis represents the most severe clinical manifestation, with reported mortality rates up to 50% [52] and clinical presentations ranging from asymptomatic troponin elevation to chest pain, dyspnea, arrhythmias, and cardiogenic shock [53].

In this context, imaging findings reflect the underlying inflammatory myocardial injury. TTE represents the first-line modality in suspected ICI-related cardiotoxicity, potentially demonstrating segmental wall motion abnormalities, increased LV wall thickness due to edema, global hypokinesia, or pericardial effusion [54]. However, LVEF is frequently preserved (up to 50% of cases), and this does not confer a better prognosis [54] (Figure 1).

A large retrospective study enrolling 101 patients with ICI myocarditis demonstrated that, despite similar baseline GLS values, only patients who developed myocarditis exhibited a significant GLS reduction (from 20.3 ± 2.6% to 14.1 ± 2.8%, *p* < 0.001), irrespective of LVEF. Importantly, GLS reduction was strongly associated with MACE, with a 4.4-fold increased risk even in patients with preserved EF [55].

The overall superiority of strain parameters over conventional echocardiography in detecting ICI-related cardiotoxicity has been confirmed in a systematic review and meta-analysis pooling together 12 studies and 554 patients [56]. The increase in myocardial stiffness and filling pressures induced by immune-mediated edema is also reflected in the deterioration of atrial reservoir strain, with average reductions of 9.0% for the left atrium (LASr) and 13.0% for the right atrium (RASr) [56]. Another study conducted in a Chinese cohort of 55 patients found a significant reduction in both TAPSE and RV GLS post-ICI treatment, revealing the impact of this therapy also on the RV [57]. The RV involvement was also confirmed by Pohl J et al. [58], demonstrating changes in both RV and RA strain, while no alteration could be found using conventional RV parameters such as TAPSE, FAC and PAPs.

CMR represents the non-invasive gold standard for myocarditis diagnosis and directly reflects the inflammatory substrate through tissue characterization. According to the updated Lake Louise criteria, diagnosis requires at least one T2-based marker of edema and one T1-based marker of injury or fibrosis [59,60] (Table 1). LGE, typically with a non-ischemic distribution, is associated with an increased risk of MACE, particularly when localized in mid-myocardial or subepicardial layers [61,62] (Figure 1). Native T1 and ECV values are often elevated, reflecting interstitial expansion, whereas T2 may be less predictive of long-term outcomes [61,62]. The expansion of the extracellular volume (ECV) reflects interstitial infiltration, with average values of 34.3% compared to 25.3% in healthy controls [63].

Despite its central role, CMR may be negative in a significant proportion of cases, highlighting the patchy nature of immune-mediated inflammation. Indeed, less than 50% of patients demonstrate LGE, and up to 40% may have completely normal scans, even in biopsy-proven myocarditis [64]. In such cases, endomyocardial biopsy remains essential [64,65]. FDG-PET may provide additional information by detecting metabolic inflammatory activity, although its routine use is not yet established [66,67].

### 2.3. Chimeric Antigen Receptor T-Cell Therapy

Chimeric antigen receptor T-cell (CAR-T) therapy is an advanced form of adoptive immunotherapy in which autologous T lymphocytes are genetically engineered to express receptors that specifically target tumor-associated antigens, enabling the direct recognition and destruction of malignant cells [68].

Cardiotoxicity associated with CAR-T therapy is primarily mediated by cytokine release syndrome (CRS), which induces a systemic inflammatory response, leading to myocardial stunning, increased vascular permeability, and hemodynamic instability [68]. This inflammatory cascade results in transient myocardial dysfunction rather than direct structural damage (Figure 1).

Clinically, cardiotoxicity manifests as tachycardia, hypotension, arrhythmias, acute heart failure, cardiogenic shock, and myocarditis-like syndromes, with an incidence of cardiovascular events exceeding 19% in patients treated for hematological malignancies [68,69].

Imaging findings reflect this dynamic and often reversible myocardial impairment. TTE is the cornerstone modality for both baseline and follow-up assessment. The most common findings include a transient reduction in LVEF, segmental wall motion abnormalities, and diastolic dysfunction with increased filling pressures, particularly in patients with severe CRS [70] (Figure 1).

Strain imaging plays a crucial role in detecting early myocardial involvement, as reductions in GLS often precede LVEF decline and are strongly associated with adverse outcomes [70,71]. Prospective data show early deterioration in GLS and left atrial reservoir strain within the first week after infusion, accompanied by an increase in the E/e′ ratio, reflecting combined systolic and diastolic dysfunction [72]. Importantly, these changes tend to be reversible within 30 days, supporting the concept of transient cytokine-mediated myocardial injury [72].

A TTE examination is recommended at baseline, during CRS with an ASTCT grade ≥ 2 (defined by the presence of fever ≥ 38.0 °C, hypotension not requiring vasopressor support and/or hypoxia requiring supplemental oxygen), and at follow-up in the case of altered symptoms or biomarkers [71,73]. Even though the exact role of CMR in this context has yet to be defined and robust data are currently lacking, some experienced centers recommend a CMR examination for LV function assessment in patients with clinical suspicion of cardiotoxicity within one month of CAR-T-cell infusion [74]. The metabolic response evaluated at FDG-PET/CT may have a prognostic role in this context; patients with relapsed or refractory (r/r) aggressive B-cell lymphomas but complete metabolic response at FDG-PET/CT after CAR T-cell treatment were more likely to still be in remission at 1-year follow-up [75].

### 2.4. Tyrosine Kinase Inhibitors

TKIs are targeted anticancer agents designed to inhibit specific intracellular signaling pathways that regulate tumor cell proliferation, angiogenesis, and survival. By blocking these kinase-driven pathways, they effectively limit tumor growth; however, their lack of complete selectivity results in off-target effects on signaling cascades that are essential for cardiovascular function [76,77].

TKIs exert cardiotoxic effects through complex off-target mechanisms, including endothelial dysfunction, impaired angiogenesis, oxidative stress, and reduced nitric oxide bioavailability [76,77] (Table 1, Figure 1). These alterations disrupt vascular homeostasis, increase afterload, and promote both myocardial dysfunction and vascular complications.

Tyrosine kinase inhibitors (TKIs) like imatinib and sorafenib induce cardiotoxicity by disrupting mitochondrial function through distinct pathways [78]. Evidence from rat heart fibers and H9c2 cells reveals that both TKIs caused mitochondrial superoxide accumulation and decreased the cellular GSH pool, inducing caspase 3/7 activation, suggesting apoptosis as a mechanism of cell death. Imatinib and sorafenib impaired the function of cardiac mitochondria in isolated rat cardiac fibers and in H9c2 cells at plasma concentrations reached in humans. Both TKIs impaired the function of the enzyme complexes of the electron transfer system (ETS), which was associated with mitochondrial ROS accumulation and cell death by apoptosis [78].

Third-generation EGFR inhibitors (e.g., osimertinib, lazertinib) carry a higher risk of cardiac events than first-generation agents, including arrhythmias, QTc prolongation, heart failure, and vascular toxicity [76].

Clinically, TKI-related cardiotoxicity encompasses a broad spectrum, including hypertension, LV dysfunction, heart failure, pulmonary hypertension, arrhythmias, Takotsubo syndrome and arterial occlusive events [77,79] (Figure 1).

Imaging findings primarily reflect vascular and hemodynamic alterations. TTE is the main modality for evaluation, allowing for the detection of LV systolic dysfunction, increased left atrial pressure, and diastolic abnormalities [80] (Table 1, Figure 1). A case of rapid aortic stenosis progression after nilotinib treatment, likely related to its in vitro capability to increase calcification and induce osteogenic activation, was described by Carracedo et al. [81]. In addition, TTE plays a key role in identifying pulmonary hypertension through the estimation of right ventricular systolic pressure [82].

Specific TKIs, such as dasatinib, can significantly elevate left atrial pressure compared to alternative agents like nilotinib and imatinib, directly correlating with the development of pulmonary effusion [80]. Furthermore, in chronic myeloid leukemia patients receiving second-generation TKIs, 4D speckle tracking echocardiography (STE) Vivid E9 ultrasound system (GE Vingmed Ultrasound) successfully identifies early subclinical myocardial dysfunction, revealing profound impairments in GLS, GCS and GAS [83]. This pattern of acute, subclinical cardiovascular alteration is highly prevalent even in pediatric and adolescent cohorts undergoing multi-targeted TKI therapy, where over 30% of patients develop new imaging evidence of cardiac dysfunction—predominantly abnormal global longitudinal strain (GLS)—alongside a high incidence of new-onset hypertension [84]. Despite these frequent functional shifts, major treatment disruptions or dose reductions remain notably rare (3.6%), suggesting that these early-stage changes are clinically manageable if caught in a timely manner [84]. Complementing these echocardiographic insights, CMR imaging in patients suspected of ibrutinib-related cardiotoxicity has revealed that nearly two-thirds exhibit signs of myocardial injury, characterized by significant post-treatment elevations in native T1 and maximum T2 mapping, as well as an increased prevalence of myocardial fibrosis identified through LGE [85]. Taken together, these findings strongly advocate for the routine integration of advanced strain imaging and parametric CMR mapping to ensure the early detection and comprehensive long-term monitoring of TKI-induced subclinical cardiotoxicity.

### 2.5. Epidermal Growth Factor Receptor-2 Inhibitors

HER2 inhibitors are targeted anticancer therapies designed to block HER2-mediated signaling pathways that drive tumor cell proliferation, survival, and resistance to apoptosis. HER2 is a transmembrane tyrosine kinase receptor belonging to the ErbB family, and its overexpression or amplification—particularly in breast cancer—is associated with aggressive tumor behavior and poor prognosis [86]. Monoclonal antibodies such as trastuzumab and pertuzumab inhibit HER2 signaling by preventing receptor dimerization and the downstream activation of key proliferative pathways, including PI3K/AKT and MAPK cascades, thereby reducing tumor growth and promoting cancer cell death [86].

However, HER2 signaling also plays a critical role in maintaining cardiomyocyte homeostasis. In the heart, the activation of the neuregulin-1 (NRG-1)/HER2 pathway is essential for cell survival, stress adaptation, and repair processes (Figure 1). The inhibition of this pathway impairs cardiomyocyte resilience, reduces the ability to respond to physiological stress, and increases susceptibility to injury [87].

As a result, cardiotoxicity associated with HER2 inhibitors is primarily mediated by the disruption of the NRG-1/HER2 signaling axis, leading to impaired cellular survival, increased oxidative stress, and mitochondrial dysfunction, ultimately resulting in contractile impairment [88] (Table 1, Figure 1).

Clinically, this translates into predominantly reversible LV systolic dysfunction (Figure 1), with an incidence of asymptomatic LVEF reduction ranging from 5% to 19% and symptomatic heart failure in up to 4% of cases, with a higher risk in the presence of previous anthracycline therapy or cardiovascular risk factors [1,89,90].

Anti-HER2 antibody–drug conjugates (ADCs) (T-DM1, trastuzumab deruxtecan) have a lower risk of cardiotoxicity than trastuzumab, with an incidence of LVEF reduction between 1% and 12% [90]. Pertuzumab, approved by the FDA for several oncological indications, shows a favorable cardiac safety profile, with heart failure rates <1% in randomized trials [90].

In this context, speckle tracking echocardiography provides incremental value, as early deterioration in global longitudinal strain (GLS ≥ 10% from baseline) predicts subsequent LVEF reduction [91] (Table 1, Figure 1). In a retrospective single-center study in 67 patients with HER2-positive breast or salivary gland cancer undergoing serial echocardiographic surveillance before and after the first, third, and sixth cycles of therapy, a relative GLS reduction > 15% from baseline was used as the trigger for the initiation or intensification of cardioprotective therapy when appropriate [92]. During follow-up, 28.4% of patients developed GLS-defined subclinical dysfunction, most cases occurring by the sixth cycle. Compared with standard management, the GLS-guided approach was associated with a significantly lower rate of trastuzumab discontinuation for cardiogenic reasons (2.4% vs. 24.0%) and a lower incidence of LVEF-based CTRCD (4.8% vs. 24.0%) while also favoring the subsequent recovery of LV function [92]. In addition to LV involvement, HER2-targeted therapies may affect other cardiac chambers, with evidence of impaired right ventricular mechanics and reduced left atrial strain in patients developing CTRCD [93,94].

CMR may offer additional tissue characterization, with small studies demonstrating subepicardial LGE in trastuzumab-induced cardiomyopathy [95] (Figure 1). Subclinical cardiotoxicity is frequent even in HER2-positive breast cancer patients at low-to-moderate cardiovascular risk, with CTRCD occurring in 25.7% of cases [96]. Early after therapy initiation, CMR detected reductions in LVEF and myocardial strain together with transient increases in native T1 and T2. Patients with baseline risk factors or anthracycline exposure showed higher T1 values, supporting T1 mapping as a sensitive marker of early myocardial injury during anti-HER2 therapy [96].

## 3. Clinical Interpretation of Imaging Findings and Surveillance Thresholds

CTRCD is diagnosed and graded primarily by changes in LVEF, GLS, and cardiac biomarkers: asymptomatic CTRCD is classified as severe when LVEF newly falls below 40%, moderate when LVEF decreases by ≥10 percentage points to 40–49% or when LVEF is 40–49% with either a relative GLS reduction >15% from baseline or new biomarker elevation, and mild when LVEF remains ≥50% but GLS falls by >15% and/or biomarkers increase [7]. In clinical practice, GLS values < 16% indicate high risk, 16–18% are borderline, and ≥18% are generally normal; a relative GLS decline ≥15% suggests early subclinical myocardial dysfunction, whereas a decline <8% makes cardiotoxicity unlikely [97,98] (Table 2). Systematic assessments of diastolic function and filling pressure remain a recommended part of routine examinations according to current guidelines [99]. Baseline echocardiography should be performed in virtually all patients before initiating cardiotoxic therapy, preferably via 3D LVEF evaluation [7]. Echocardiographic follow-up should be tailored to baseline risk and treatment type: for anthracyclines, imaging is recommended at baseline and according to risk, with closer monitoring at the second, fourth, and sixth cycles; 3 months; and 1 year in high-risk patients and additional evaluation once the cumulative doxorubicin-equivalent dose exceeds 250 mg/m^2^; after treatment, follow-up is advised at completion and at 6 and 12 months [98,99,100] (Table 2). For anti-HER2 therapy, surveillance is generally performed at baseline and every 3 months during treatment, while for VEGF/angiogenesis inhibitors, it is risk-adapted, usually every 4 months in low/moderate-risk patients and every 3 months in high-risk patients, with earlier reassessment in very-high-risk cases [98] (Table 2). For immune checkpoint inhibitors, no echo-specific cut-off is established; myocarditis should be suspected in the presence of troponin elevation with compatible clinical, ECG, CMR, or echocardiographic findings, and early GLS reduction may support detection, particularly within the first 3 months of therapy [7,101] (Table 2). After the completion of systemic therapy, patients exposed to anthracyclines should undergo echocardiography at the end of treatment and at 6 and 12 months, whereas follow-up after non-anthracycline regimens may stop if post-treatment imaging is normal [98]. Radiotherapy-induced heart disease (RIHD) is considered a distinct entity, with no dedicated LVEF/GLS diagnostic threshold; risk stratification is based mainly on mean heart dose, cumulative anthracycline exposure, left/anterior chest irradiation, total dose > 30 Gy, fraction dose > 2 Gy/day, young age, pre-existing cardiovascular disease, and conventional risk factors [16] (Table 2). Because RIHD may emerge 5–10 years or even decades after exposure, periodic long-term echocardiographic surveillance is recommended in moderate-, high-, and very-high-risk survivors, with earlier assessment if symptoms or suspected structural, ischemic, pericardial, valvular, or pulmonary pressure abnormalities occur [7,102] (Table 2). Multimodality approaches to diagnosing CTRCD involve CMR-based tissue characterization, which establishes a validated subclinical diagnostic threshold through quantitative mapping algorithms: early myocardial involvement is defined by a progressive elongation in both native T1 and T2 relaxation times exceeding ≥2 standard deviations (SDs) from normal reference baselines, whereas chronic subclinical changes shift to permanently elevated native T1 values ≥ 2SD alongside normalized T2 times, either with or without a concomitant impairment in global longitudinal strain (GLS < 17%) [54,103] (Table 2). Furthermore, subclinical deteriorations in CMR global circumferential strain (GCS) have emerged as robust, geometry-independent predictors of early-stage toxicity, which can be further supported by an absolute extracellular volume (ECV) expansion threshold of ≥29% reflecting diffuse interstitial fibrosis [104]. Although CCTA’s role remains primarily focused on ruling out coronary artery disease in low-to-intermediate-risk patients presenting with a newly reduced LVEF < 50%, modern non-contrast CT applications utilize an Agatston Calcium Score baseline assessment to stratify long-term major adverse cardiovascular event (MACE) risk [105] (Table 2). Concurrently, emerging dual-energy CT protocols are being investigated to quantify CCTA-derived myocardial ECV, demonstrating strong diagnostic correlation with CMR and histological fibrosis to comprehensively integrate standard echocardiographic workflows [106].

## 4. Cardiac Effects of Radiotherapy: Mechanisms and Imaging Findings

**Figure 2:**
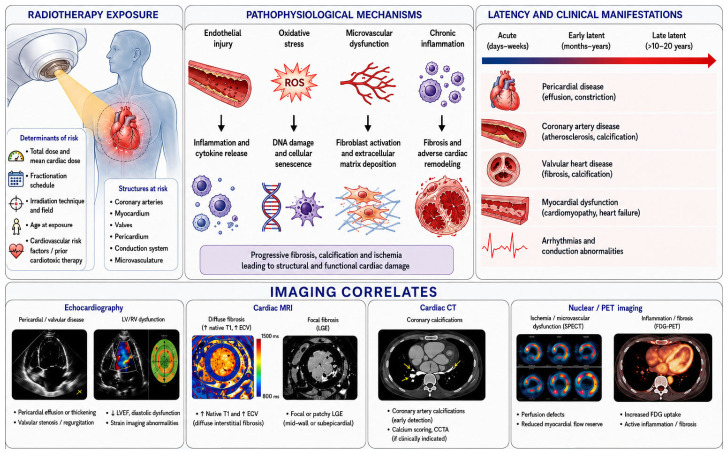
Regional myocardial injury patterns and advanced imaging biomarkers in radiotherapy-induced cardiotoxicity. Radiation exposure may induce heterogeneous and region-specific myocardial injury through endothelial dysfunction, chronic inflammation, oxidative stress, and fibrosis. Advanced deformation imaging techniques, including regional and principal strain analysis, may identify localized subclinical dysfunction corresponding to irradiated myocardial regions before global ventricular impairment develops.

Radiotherapy (RT) can also induce cardiotoxicity, with clinical manifestations occurring both acutely and decades after treatment [107,108] (Figure 2). The risk is strongly influenced by total dose, fractionation, irradiation techniques, age at exposure, and the presence of cardiovascular comorbidities or concomitant cardiotoxic therapies [109,110,111]. Epidemiological studies have demonstrated a linear dose–response relationship, with a 7.4% increase in major cardiac events for each Gray of mean cardiac dose in breast cancer patients [111,112].

RT-related cardiotoxicity encompasses a broad spectrum of conditions, including pericardial disease, coronary artery disease, valvular heart disease, myocardial dysfunction, and arrhythmias, often presenting years after exposure with non-specific symptoms such as dyspnea, chest pain, or arrhythmias [111].

### 4.1. Molecular and Inflammatory Mechanisms

Radiotherapy-induced cardiotoxicity is driven by endothelial dysfunction, oxidative stress, chronic inflammation, and progressive microvascular injury with the NLRP3 inflammasome emerging as a central molecular hub [113] (Figure 2). Ionizing radiation induces direct DNA damage and ROS generation, activating ATM/NF-kB signaling and promoting pro-inflammatory mediators, including NLRP3, pro-IL-1beta, and pro-IL-18 [113,114]. Mitochondrial dysfunction further amplifies redox stress and inflammasome activation, while impaired mitochondrial quality control and cell death pathways sustain DAMP release and chronic innate immune signaling. Additional upstream signals include K+ efflux, Ca2+ flux, lysosomal destabilization, cholesterol crystal formation, and ceramide accumulation, all of which further contribute to NLRP3 activation [115,116]. Impaired mitochondrial quality control, particularly altered mitophagy and fusion–fission dynamics, sustains a feed-forward cycle of oxidative stress, while apoptosis, necroptosis, ferroptosis, and pyroptosis release DAMPs that reinforce chronic innate immune activation [113]. Overall, persistent inflammasome activity links radiation exposure to fibrosis, vascular damage, and electrophysiological remodeling.

### 4.2. RT Protocols and Dose–Response Relationships

Modern RT techniques have reduced overall cardiac exposure, but the risk of cardiotoxicity remains strongly influenced by spatial dose distribution (a). Compared with 3DCRT, intensity-modulated radiotherapy (IMRT) and volumetric modulated arc therapy (VMAT) improve dose conformity and reduce high-dose exposure to cardiac substructures such as the LAD and LV, although often at the expense of greater low-dose spread to surrounding tissues. Conversely, intensity-modulated radiotherapy (IMRT) and volumetric modulated arc therapy (VMAT) improve dose conformity and reduce high-dose volumes to the LAD and LV but may increase very-low-dose volumes delivered to the lungs and the contralateral breast, highlighting a distinct trade-off between conformity and the “low-dose bath” [117,118]. In the context of whole-breast RT with deep-inspiration breath-hold (DIBH), tangential VMAT (tVMAT) offers superior homogeneity and conformity compared to field-in-field 3DCRT. It achieves this while maintaining comparable mean doses to the heart, LAD, and LV and reducing contralateral “spillover” relative to continuous-arc VMAT, all with moderate monitor unit requirements [119]. More broadly, incorporating angular complexity (such as short-arc VMAT or multi-beam IMRT) allows for a reduction in high doses to the LAD and LV (e.g., LAD V40/V30, LV V23) compared to traditional tangential fields. Meanwhile, 3DCRT often remains more favorable for very-low-dose volumes, such as LV V5, reinforcing the inherent balance between high-dose sparing and low-dose spreading [118]. When DIBH is not feasible, 4DCT-based robust IMRT/VMAT planning represents a valid alternative, mainly by limiting LAD hotspots. In pediatric and long-term survivor settings, proton therapy provides the lowest mean doses to the heart and other organs at risk, supporting individualized modality selection to minimize late cardiovascular effects [120]. Finally, hybrid approaches that integrate field-in-field techniques with IMRT or VMAT, including post-mastectomy regimens, improve conformity and limit low-dose contralateral spreading compared to “pure” IMRT or VMAT while maintaining adequate planning efficiency [117].

### 4.3. RT-Related Cardiotoxicity

Despite technological improvements in treatment planning and delivery conformality, radiation-induced heart disease (RIHD)—encompassing arrhythmia, heart failure, and ischemic events—affects approximately 20% of patients [121]. The clinical relevance of this toxicity has been highlighted by landmark trials such as RTOG-0617 and LungART, where higher cardiac radiation doses were significantly correlated with worse overall survival, establishing heart dose as a critical therapeutic limiting factor [121]. The long-term results of the phase III randomized NRG Oncology RTOG 0617 trial evaluated standard-dose (SD; 60 Gy) versus high-dose (HD; 74 Gy) radiation therapy (RT) with concurrent chemotherapy, with or without cetuximab, demonstrating that SD RT provided a significantly superior median overall survival (OS) compared to HD RT (28.7 versus 20.3 months, *p* = 0.0072), alongside a significantly higher 5-year OS rate (32.1% versus 23.0%) [122]. Multivariable analysis confirmed that standard radiation dose, smaller planning target volumes, tumor location, higher institution accrual volume, and reduced heart radiation doses (heart V5/V30) were significantly associated with improved OS [122]. Similarly, the LungART trial demonstrated that despite improving local disease control, postoperative radiation exposure carried severe cardiopulmonary toxicity that directly negated overall survival benefits [123]. When radiation fields disproportionately impact specific sub-regions, the downstream functional consequences diverge systematically: excess doses to the atria and pulmonary veins trigger acute electrical remodeling and arrhythmias like atrial fibrillation, whereas targeted exposure to the coronary arteries and left ventricle provokes accelerated atherosclerosis, ischemic myocardial infarction, and congestive heart failure [121].

### 4.4. Translational Implications: Preclinical vs. Clinical Outcomes

Preclinical and clinical studies converge in demonstrating that myocardial strain imaging detects radiation-induced cardiotoxicity at an early stage, when the left ventricular ejection fraction (LVEF) is still preserved. In murine models undergoing partial or total heart irradiation, early, regional, and dose-dependent CMR alterations in strain manifest as early as 4 to 10 weeks as long-term predictors of systolic–diastolic dysfunction, remodeling, and diffuse fibrosis [124,125,126,127]. Strain maps reveal a heterogeneous decline that is more pronounced in segments receiving higher doses, which correlates histologically with vacuolization, inflammatory infiltrates, necrosis, and, notably, capillary rarefaction and reduced microvascular density [128]. Clinically, prospective studies in breast cancer patients undergoing RT document a subclinical reduction in GLS in approximately 12–14% of cases within 6 months, even while conventional echocardiographic parameters and LVEF remain substantially stable (Figure 2). This risk increases with higher mean heart doses and larger low-dose left ventricular volumes (e.g., LV V5, LV*_Dmean_* > 3 Gy), confirming a clear dose–response relationship [129,130]. The consistency between animal and human data—including the initial preservation of LVEF and the chronological precedence of regional strain alterations over global markers—strongly supports the use of speckle tracking and advanced cardiovascular magnetic resonance (CMR) as sensitive tools for the early identification of radiation-induced myocardial injury.

CMR parametric mapping techniques enable the detection of diffuse interstitial fibrosis, with increased native T1 and ECV observed in irradiated patients compared to controls, even in the absence of focal LGE [131,132] (Figure 2). However, these alterations appear attenuated with modern low-dose radiotherapy techniques, suggesting a reduced structural impact compared with historical cohorts [133].

Cardiac CT is particularly sensitive for detecting radiation-induced coronary calcifications, although its use in surveillance is limited by cumulative radiation exposure, especially in younger patients [70]. Current guidelines recommend baseline echocardiographic assessment in high-risk patients prior to RT, with CMR reserved for cases with inconclusive findings or suspected structural abnormalities [8,70].

## 5. Age-Related Differences in Cardiotoxicity

Cardiotoxicity is not limited to adults. Childhood cancer survivors experience a markedly increased lifetime cardiovascular burden, with up to 21.6% developing cardiomyopathy and 83.5% valvular disease by the age of 50, making cardiovascular disease the leading noncancer cause of death in this population [134,135]. Unlike adults, in whom cardiotoxicity often manifests during or shortly after treatment, pediatric patients are exposed to a substantially higher cumulative lifetime risk, with cardiac complications that may emerge years or decades after therapy and continue to increase over time without reaching a clear plateau [136,137,138,139] (Table 3).

This increased vulnerability reflects fundamental developmental differences. The pediatric myocardium is structurally immature and more susceptible to apoptosis, with a reduced capacity to compensate for cellular injury [140,141] (Table 3). Exposure to cardiotoxic therapies during critical phases of growth interferes with myocardial maturation, limiting long-term adaptive reserve and, in some cases, resulting in reduced cardiac mass and impaired functional capacity, a phenotype described as “Grinch syndrome” [142]. In addition, pharmacokinetic differences further contribute to susceptibility, particularly in infants and young children, who exhibit reduced clearance of anthracyclines and therefore higher effective exposure even at lower administered doses [143,144,145] (Table 3). Consistently, structural abnormalities such as reduced LV wall thickness, decreased LV mass, and adverse remodeling have been documented even at relatively low cumulative doses, supporting the concept that no truly safe dose exists for the developing heart [141].

A key feature of pediatric cardiotoxicity is the high prevalence of subclinical myocardial dysfunction. As in adults, early cardiac injury may remain undetected if surveillance relies solely on conventional parameters such as LVEF. For this reason, multimodal approaches integrating advanced echocardiographic indices—particularly myocardial strain and diastolic parameters—with electrocardiographic findings and circulating biomarkers are increasingly being investigated to improve early detection and long-term risk stratification [146]. In pediatric patients, this approach is particularly relevant, as even subtle abnormalities detected during childhood may have long-term clinical implications when combined with future physiological stressors such as pregnancy, hypertension, or metabolic disease [70,142,147].

These pathophysiological and temporal differences translate into distinct clinical priorities for cardiovascular imaging and surveillance. In adults, imaging strategies are primarily focused on baseline risk assessment and early detection of treatment-related dysfunction during active therapy, with particular attention to acute complications. In contrast, pediatric imaging must provide a lifelong reference framework, as most patients begin treatment without pre-existing cardiovascular disease. Baseline imaging therefore acquires particular importance, serving as a comparator for longitudinal evaluation over decades [140,141,148].

Given the progressive and often delayed nature of cardiotoxicity in this population, surveillance must be structured, risk-adapted, and lifelong. Current recommendations suggest echocardiographic follow-up every two years in high-risk survivors and every five years in moderate-risk individuals, with adjustments based on clinical status and treatment exposure [97] (Table 3). Importantly, the consistency of imaging techniques and reproducibility of measurements are essential, particularly during the transition from pediatric to adult care, to ensure accurate longitudinal comparisons [140,146,149].

Imaging in pediatric cardio-oncology also presents specific technical challenges. The continuous changes in cardiac size, geometry, and physiology during growth require the use of age- and size-adjusted reference values, while longitudinal assessment may be complicated by limited reproducibility across different stages of development [110]. In this context, myocardial strain analysis has emerged as a particularly valuable tool, offering greater sensitivity for detecting subtle myocardial dysfunction and improved reproducibility compared with conventional measures such as LVEF [70,141,150] (Table 3).

CMR is widely used to characterize myocardial tissue subclinical alterations in children; however, its use in younger children is often limited by the need for sedation or general anesthesia, longer acquisition times, and challenges related to patient cooperation [151,152,153]. As a result, CMR is generally reserved for selected cases, such as suboptimal echocardiographic windows or borderline findings, in accordance with current ESC recommendations [1].

Although cardiac CT and positron emission tomography (PET) can provide valuable information in specific clinical scenarios, their use in pediatric patients is limited by concerns related to radiation exposure and contrast administration. In children, thoracic RT is a known risk factor for late CVD, but doses < 10 Gy do not significantly increase the risk of late heart disease, although the pediatric population is exposed to a higher cumulative risk due to their long post-treatment life expectancy (Table 3). The PENTEC review highlights that for every 10 Gy of mean cardiac dose, the risk of coronary artery disease, heart failure, and valvular heart disease doubles in pediatric survivors [112]. Consequently, echocardiography and CMR remain the cornerstone imaging modalities in pediatric cardio-oncology [141]. In contrast, these techniques may become more relevant later in adulthood, particularly in survivors exposed to mediastinal radiotherapy, in whom coronary artery calcium scoring and CT angiography can aid in the detection of radiation-induced coronary artery disease [105]. Similarly, PET imaging, rarely used during childhood follow-up, may gain a role in adulthood for the evaluation of myocardial ischemia and microvascular dysfunction [154].

Long-term management extends beyond imaging and includes the need for a structured transition from pediatric to adult care. This process should be initiated early in survivorship and supported by patient education, clear communication, and the coordinated transfer of clinical information. Individualized transition plans based on treatment exposure, cumulative doses, and current cardiac status are essential to ensure continuity of care and adherence to surveillance strategies [155,156].

Finally, long-term care should also incorporate active strategies to reduce cardiovascular risk. Lifestyle interventions, including dietary counseling, blood pressure control, and weight management, are particularly important in this high-risk population [142,156,157]. Exercise prescription represents a key component of preventive care, with recommendations including regular moderate-intensity aerobic activity combined with strength training, tailored to individual functional capacity and treatment-related limitations [158,159]. Supervised programs led by clinicians or exercise specialists with expertise in cardio-oncology are preferred, as they have demonstrated greater effectiveness in improving cardiopulmonary fitness and long-term adherence [160,161,162].

**Table 3:** A comparison of cardiotoxicity mechanisms, structure–function relationships, clinical outcomes and imaging surveillance implications between adults and children/AYA cancer survivors. This table summarizes the distinct differences in cancer therapy-related cardiovascular toxicity between adult patients and pediatric or adolescent and young adult (AYA) cancer survivors. The discrepancies are categorized across four core domains: pathophysiological and pharmacokinetic mechanisms (e.g., myocardial substrate and anthracycline clearance), structure–function phenotypes characterized by advanced imaging (CMR and GLS), long-term clinical burden and radiation dose–response relationships, and the subsequent divergent strategies required for long-term imaging and surveillance.

Aspect	Adults	Children and AYA Cancer Survivors	Key Studies
Mechanisms			
Myocardial substrate	Mature myocardium with greater compensatory and reparative reserve	Immature, still-developing myocardium; higher susceptibility to apoptosis and reduced myocardial reserve	Bennati [140]; Mertens [141]
Anthracycline pharmacokinetics	Normal clearance	Reduced clearance, particularly in infants and young children, yielding higher effective exposure even at lower administered doses; no truly “safe” dose for the developing heart	Leger [143]; Johnson [144]; Sieswerda [145]
Dominant injury pattern	Dose-dependent, largely irreversible anthracycline (type I) injury via oxidative/mitochondrial damage; therapy-specific patterns for HER2, VEGF and immune checkpoint inhibitors	Disruption of myocardial maturation and growth, leading to reduced cardiac size and contractile reserve (“Grinch syndrome”)	Lipshultz [142]
Structure-function relationships			
Remodeling phenotype	Replacement fibrosis, adverse LV remodeling and progression to overt heart failure	Impaired growth with reduced LV wall thickness and mass even at low cumulative doses, with progressive subclinical remodeling	Jafari [23]; Mertens [141]; Leger [143]
Tissue characterization (CMR)	Diffuse fibrosis and extracellular-volume expansion detectable by parametric mapping before LVEF decline	Diffuse interstitial fibrosis on T1 mapping correlates with cumulative dose and remodeling; progressive ECV rise demonstrated after anthracyclines	Meléndez [153]
Deformation imaging (GLS)	Sensitive marker of subclinical dysfunction that precedes LVEF fall	Highly sensitive and more reproducible than LVEF; a core screening parameter that must remain consistent across growth	Niemelä [149]; Mihos [150]
Clinical outcomes			
Temporal pattern	Frequently early/acute, during therapy or within the first year	Lifelong and progressive; cumulative incidence rises 5–10 years after exposure without an apparent plateau	Mulrooney [138]; Tukenova [139]
Long-term burden	Largely driven by pre-existing cardiovascular risk factors and concomitant therapies	By age 50, up to ≈21.6% develop cardiomyopathy and ≈83.5% valvular disease; cardiovascular disease is the leading non-cancer cause of death	Brickler [134]; Hudson [135]; Hammoud [147]
Radiation dose-response	Breast radiotherapy: ≈7.4% increase in major cardiac events per Gy of mean heart dose	Every 10 Gy of mean heart dose doubles the risk of coronary disease, heart failure and valvular disease; doses <10 Gy add little early risk but act over a far longer life expectancy	Ell [111]; Bates–PENTEC [112]
Imaging and surveillance implications			
Primary aim	Pre-treatment risk stratification and early detection during active therapy	Lifelong surveillance anchored to a baseline reference, with continuity across growth and transition to adult care	Bennati [140]; Chow [148]
Modality constraints	CMR, CT and PET broadly feasible; CT/PET useful for radiation-related coronary disease	CMR frequently requires sedation; CT/PET constrained by radiation exposure, so echocardiography with strain remains the core modality	Scialò [151]; Lopez-Mattei [105]; Milanesi [154]
Surveillance schedule	Most intensive during treatment, risk-adapted thereafter	Biennial echocardiography in high-risk and every 5 years in moderate-risk survivors (AHA–Children’s Oncology Group)	Ehrhardt [136]; Ryan [137]; Armenian [157]

Abbreviations: AHA, American Heart Association; AYA, adolescent and young adult; CMR, cardiac magnetic resonance; CT, computed tomography; ECV, extracellular volume; GLS, global longitudinal strain; Gy, gray; HER2, human epidermal growth factor receptor 2; LV, left ventricle; LVEF, left ventricular ejection fraction; PET, positron emission tomography; VEGF, vascular endothelial growth factor.

## 6. Future Directions and AI

In recent years, artificial intelligence (AI) and machine learning (ML) have been driving personalized risk stratification for CTR-CVT [163,164,165,166]. The methodological foundation for this transition stems from computational advances in general cardiology and surgery, where models overcome the limits of traditional linear risk calculators (EuroSCORE II/STS) [167]. A key example is multi-fidelity learning applied to myocardial infarction, which combines rodent-based in silico simulations with limited human data [168]. This framework allows UNet neural networks to quantify infarcted tissue using only cardiac strain maps, offering a contrast-free alternative to LGE-CMR [168]. Parallelly, these algorithms are transitioning into real-world clinical implementation, translating perioperative frameworks and surgical computer vision directly into cardio-oncology surveillance [167]. In echocardiography, AI enables the automated and reproducible quantification of LVEF and GLS during cardiotoxic regimens (e.g., trastuzumab) [169]. In CMR workflows, deep learning optimizes volumetric segmentation, strain feature tracking, and parametric T1/T2 mapping, intercepting latent edema from immune checkpoint inhibitors (ICIs) or diffuse fibrosis [164]. Furthermore, machine learning applied to pretreatment cardiovascular CT scans extracts advanced texture features from cardiac substructures and the thoracic aorta to predict radiation-induced toxicity, guiding preventive dosimetry [170]. Artificial intelligence (AI) is emerging as a promising tool for enhancing follow-up care in childhood cancer survivors by supporting cardiovascular surveillance across the lifespan [166,171]. Early studies show that AI models have demonstrated the ability to integrate complex data, including clinical history, treatment exposures, risk factors, and genetic data, to predict a range of cardiovascular outcomes [163,164,165,166]. However, it should be noted that so far, AI models have undergone only internal validation, resulting in a high risk of bias and therefore limiting their clinical applicability. Consequently, the future of the discipline lies in the rigorous consolidation of hybrid and multimodal models capable of combining biophysical simulations with real-world clinical datasets while ensuring standardized, prospective, and multicenter clinical validation.

## 7. Conclusions

Cancer therapy-related cardiotoxicity is an increasingly important clinical challenge in both adult and pediatric populations, reflecting the growing number of long-term cancer survivors. In this context, multimodality imaging plays a central role in early detection, risk stratification, and longitudinal monitoring, enabling the identification of subclinical dysfunction before overt disease develops. Advanced imaging techniques have improved the detection of early myocardial injury beyond conventional parameters.

Pediatric patients represent a uniquely vulnerable population due to their myocardial immaturity and long life expectancy. This highlights the need for dedicated lifelong surveillance strategies and a structured transition to adult care.

In conclusion, integrating multimodality imaging with mechanistic understanding and emerging technologies is essential to improve early detection and long-term cardiovascular outcomes, particularly in pediatric cancer survivors.

## Figures and Tables

**Table 1:** Mechanisms, cardiotoxic effects, and multimodality imaging findings associated with major classes of antineoplastic therapies.

Antineoplastic Drug	Cardiotoxic Effects	Mechanisms	Imaging Findings/Recommended Modality
Anthracyclines	LV dysfunction/HF; cardiomyopathy; arrhythmias; SCD	DNA damage; mitochondrial dysfunction; oxidative stress; cardiomyocyte apoptosis	TTE: LVEF decline; GLS reduction (early). CMR: ↑T1, ↑ECV, fibrosis
HER2 inhibitors	LV dysfunction/HF; cardiomyopathy	NRG-1/HER2 pathway inhibition; impaired repair; oxidative stress	TTE: LVEF reduction; GLS early decline; serial monitoring
VEGF inhibitors	LV dysfunction; ischemia; hypertension; thrombosis; TTS	Impaired angiogenesis; NO depletion; vasoconstriction; oxidative stress	TTE: LV dysfunction; stress imaging if ischemia suspected
TKIs	LV dysfunction; arrhythmias; QT prolongation; ischemia	Endothelial dysfunction; NO depletion; oxidative stress	TTE: LV dysfunction; filling pressures; RVSP
ICIs	Myocarditis; arrhythmias; pericarditis; ischemia	T-cell activation; inflammation; autoimmunity	TTE: often normal EF; GLS reduction. CMR: Lake Louise criteria

Each class of anticancer agents is characterized by specific pathophysiological mechanisms leading to distinct patterns of cardiotoxicity. Multimodality imaging, including transthoracic echocardiography (TTE), speckle tracking echocardiography (GLS), and cardiac magnetic resonance (CMR), plays a central role in the early detection, risk stratification, and longitudinal monitoring of therapy-related cardiac dysfunction. Abbreviations: HF, heart failure; GLS, global longitudinal strain; CMR, cardiac magnetic resonance; LVEF, left ventricular ejection fraction; ECV, extracellular volumeDNA: deoxyribonucleic acid; SCD: sudden cardiac death; HER2: human epidermal growth factor receptor 2; HF: heart failure; LV: left ventricle; NRG-1: neuregulin-1; NO: Nitric oxide; TKIs: Tyrosine Kinase Inhibitors; ICIs: Immune Checkpoint Inhibitors; RVSP: Right Ventricular Systolic Pressure.

**Table 2:** Imaging thresholds and surveillance strategies for cancer therapy-related cardiovascular toxicity across major anticancer therapies. This table summarizes the main imaging-based thresholds and surveillance strategies currently used for the early detection and follow-up of cancer therapy-related cardiovascular toxicity (CTR-CVT). It integrates echocardiographic criteria for asymptomatic CTRCD grading, GLS-based risk stratification, the complementary role of CMR tissue characterization, and the selective use of cardiac CT/CCTA, together with treatment-specific surveillance recommendations for anthracyclines, anti-HER2 agents, VEGF/angiogenesis inhibitors, tyrosine kinase inhibitors, immune checkpoint inhibitors, and radiotherapy-associated cardiotoxicity.

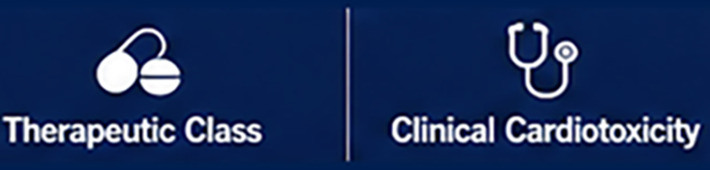	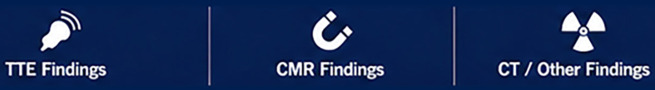	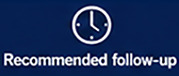
Asymptomatic CTRCD grading	Severe: LVEF < 40% Moderate: LVEF fall ≥ 10% to 40–49%, or 40–49% + GLS fall > 15%/cardiac biomarkers’ rise Mild: LVEF ≥ 50% + GLS fall > 15% and/or cardiac biomarkers’ rise	Repeat echo (moderate: within 2–3 wks); start/optimize cardioprotection; hold agent if severe (drug-/severity-dependent); CMR if uncertain or discordant
GLS risk stratification	GLS < 16% high · 16–18% borderline · ≥18% normal; relative fall ≥ 15% = subclinical; fall < 8% = CTRCD unlikely	Same vendor/software; always compare with baseline
CMR tissue characterization	Early: native T1 & T2 prolonged >2 SD. Chronic: native T1 prolonged >2 SD, normal T2 ± GLS < 17%. ECV ≥ 29% = diffuse fibrosis;	When echo inconclusive, for fibrosis/tissue characterization, or serial subclinical monitoring; detects injury before LVEF decline
Cardiac CT/CCTA	Rule out CAD if new LVEF < 50% (low–intermediate risk); dual-energy CT estimates myocardial ECV	Not for routine serial follow-up; targeted coronary assessment
Agent-specific surveillance		
Anthracyclines	General CTRCD thresholds; cumulative doxorubicin-equiv > 250 mg/m^2^ increases risk	Baseline: Low/mod risk: cycle 4 + 1 yr. High risk: cycles 2/4/6, 3 mo, 1 yr. Post-therapy: end + 6 & 12 mo. CMR if equivocal
Anti-HER2 (e.g., trastuzumab)	CTRCD LVEF/GLS criteria; early GLS fall ≥ 10% predicts later LVEF decline	Baseline + every 3 mo during therapy; CMR if borderline
VEGF/angiogenesis inhibitors	Risk-adapted; LV dysfunction, hypertension, thromboembolism	Baseline: every 4 mo (low/mod), every 3 mo (high); early echo ~3 wk if very high risk
Tyrosine kinase inhibitors (TKIs)	No specific LVEF/GLS cut-off; Higher risk with 2nd/3rd-generation TKIs (dasatinib, ponatinib); Bruton TKI associated with atrial fibrillation	Serial echo, esp. high-risk and 2nd/3rd-gen TKIs. Dasatinib/ponatinib (high/very-high risk): every 3 mo in first year and then every 6–12 mo.
Immune checkpoint inhibitors (ICIs)	No echo-only cut-off; myocarditis = cardiac biomarkers’ rise + clinical/ECG/CMR/echo; early (<3 months) GLS < 15%	Baseline if high-risk; prompt echo ± CMR on symptoms/cardiac biomarkers’ rise/ECG change/suspected myocarditis (esp. first 3 mo); CMR = modified Lake Louise criteria
Radiotherapy—associated cardiotoxicity		
Radiation- induced heart disease	No specific LVEF/GLS cut-off; risk rises with mean heart dose, anthracycline dose, left/anterior RT, total > 30 Gy, fraction > 2 Gy/day, young age, CVD	Long-term risk-adapted; periodic echo in mod/high-risk survivors; echo ± CMR for symptoms or suspected pericardial/valvular/myocardial/coronary/conduction disease
High-risk RIHD phenotype	Mediastinal/left-breast RT, RT + anthracyclines, young survivors, pre-existing CVD	Surveillance beyond 5–10 yr, possibly decades; CMR for suspected fibrosis/pericardial disease

Abbreviations: CAD, coronary artery disease; CCTA, coronary CT angiography; CMR, cardiac magnetic resonance; CT, computed tomography; CTRCD, cancer therapy–related cardiac dysfunction; CTR-CVT, cancer therapy–related cardiovascular toxicity; CVD, cardiovascular disease; ECG, electrocardiogram; ECV, extracellular volume; GCS, global circumferential strain; GLS, global longitudinal strain; Gy, gray; HER2, human epidermal growth factor receptor 2; LV, left ventricle; LVEF, left ventricular ejection fraction; RIHD, radiation-induced heart disease; RT, radiotherapy; SD, standard deviation; STE, speckle-tracking echocardiography; TTE, transthoracic echocardiography; VEGF, vascular endothelial growth factor.

## Data Availability

No new data were created or analyzed in this study.

## Abbreviations

The following abbreviations are used in this manuscript:

Ach	acetylcholine
ATP	adenosine triphosphate
CAD	coronary artery disease
CAR-T	chimeric antigen receptor T-cell
CMR	cardiac magnetic resonance
CT	computed tomography
CTLA-4	cytotoxic T-lymphocyte antigen 4
CTR-CVT	cancer therapy-related cardiovascular toxicity
CTRCD	cancer therapy-related cardiac dysfunction
ECG	electrocardiogram
ECV	extracellular volume
EGFR	epidermal growth factor receptor
ERK	extracellular signal-regulated kinase
ESC	European Society of Cardiology
FDG	fluorodeoxyglucose
GLS	global longitudinal strain
HER2	human epidermal growth factor receptor-2
HF	heart failure
HFA-ICOS	Heart Failure Association–International Cardio-Oncology Society
ICI	immune checkpoint inhibitor
IL	interleukin
LAG-3	lymphocyte activation gene 3
LGE	late gadolinium enhancement
LV	left ventricle
LVEF	left ventricular ejection fraction
NO	nitric oxide
NRG-1	neuregulin-1
NLRP3	NLR family pyrin domain-containing 3
PD-1	programmed cell death protein 1
PD-L1	programmed death ligand 1
PET	positron emission tomography
QTc	corrected QT interval
ROS	reactive oxygen species
RT	radiotherapy
RV	right ventricle
SCD	sudden cardiac death
TKI	tyrosine kinase inhibitor
TTE	transthoracic echocardiography
TTS	Takotsubo syndrome
VEGF	vascular endothelial growth factor
